# Review of Weed Detection Methods Based on Computer Vision

**DOI:** 10.3390/s21113647

**Published:** 2021-05-24

**Authors:** Zhangnan Wu, Yajun Chen, Bo Zhao, Xiaobing Kang, Yuanyuan Ding

**Affiliations:** 1 Department of Information Science, Xi’an University of Technology, Xi’an 710048, China; 2190820003@stu.xaut.edu.cn (Z.W.); kangxb@xaut.edu.cn (X.K.); 2190820010@stu.xaut.edu.cn (Y.D.); 2 Chinese Academy of Agricultural Mechanization Sciences, Beijing 100083, China; zhaoboshi@126.com

**Keywords:** weed detection, computer vision, image processing, deep learning, machine learning

## Abstract

Weeds are one of the most important factors affecting agricultural production. The waste and pollution of farmland ecological environment caused by full-coverage chemical herbicide spraying are becoming increasingly evident. With the continuous improvement in the agricultural production level, accurately distinguishing crops from weeds and achieving precise spraying only for weeds are important. However, precise spraying depends on accurately identifying and locating weeds and crops. In recent years, some scholars have used various computer vision methods to achieve this purpose. This review elaborates the two aspects of using traditional image-processing methods and deep learning-based methods to solve weed detection problems. It provides an overview of various methods for weed detection in recent years, analyzes the advantages and disadvantages of existing methods, and introduces several related plant leaves, weed datasets, and weeding machinery. Lastly, the problems and difficulties of the existing weed detection methods are analyzed, and the development trend of future research is prospected.

## 1. Introduction

At present, many smart agriculture tasks, such as plant disease detection, crop yield prediction, species identification, weed detection, and water and soil conservation, are realized through computer vision technology [1,2,3]. Weed control is an important means to improve crop productivity. Considerable literature has proposed precise variable spraying methods to prevent waste and herbicide residual problems caused by the traditional full-coverage spraying [4]. To achieve precise variable spraying, a key issue that should be solved is how to realize real-time precise detection and identification of crops and weeds.

Methods for realizing field weed detection by using computer vision technology mainly include traditional image processing and deep learning. When weed detection is conducted with traditional image-processing technology, extracting features, such as color, texture, and shape, of the image and combining with traditional machine learning methods, such as random forest or Support Vector Machine (SVM) algorithm, for weed identification are necessary [5]. These methods need to design features manually and have high dependence on image acquisition methods, preprocessing methods, and the quality of feature extraction. With the improvement in computing power and the increase in data volume, deep learning algorithms can extract multiscale and multidimensional spatial semantic feature information of weeds through Convolutional Neural Networks (CNNs) due to their enhanced data expression capabilities for images, avoiding the disadvantages of traditional extraction methods. Therefore, they have attracted increasing attention from researchers.

Several reviews on the application of machine learning in agriculture [6] and an overview of using deep learning methods to achieve agricultural tasks have been presented [7]. They have either provided a comprehensive overview of the methods applied in the entire agricultural field [8] or conducted the latest research on a certain type of technology for a specific task [9]. For example, Koirala et al. [10] summarized the application of deep learning in fruit detection and yield estimation, including the problem and solution to fruit being occluded in imaging. However, they focused only on detection and yield estimation and disregarded other agricultural tasks that contain a large number of objects, such as weed detection. Kamilaris et al. [7] reviewed the application of deep learning in agriculture, involving many studies in the fields of weed identification, land cover classification, plant identification, fruit counting, and crop type classification. Nevertheless, it was only a summary of the current situation in weed detection. Yuan et al. [11] elucidated the research progress in field weed identification at home and abroad and the advantages and disadvantages of various segmentation, extraction, and identification methods. Nonetheless, few discussions were presented about the use of deep learning methods to solve the problem of weed identification. Hasan et al. [12] provided a comprehensive review of weed detection and classification research but focused on methods based on deep learning.

Traditional and deep learning-based weed detection methods have their own advantages. Traditional weed detection methods require small sample sizes, have low requirements on graphics processing units, and can be used in agricultural machinery and equipment at a low cost. This paper mainly reviews the related methods for weed detection in recent years from the perspectives of traditional machine learning (ML) methods and deep learning and briefly discusses the pros and cons of the methods. The datasets of weed identification and detection and leaf classification are summarized, and the problems faced in field weed detection under different conditions are analyzed. This paper provides a certain reference to other scholars to further their research on weed detection algorithms based on computer vision and achieve intelligent weed control and related areas of research and application.

## 2. Public Image Datasets

Many public and annotated image datasets are available in the field of computer vision, such as ImageNet [13], COCO [14], Pascal VOC [15], and Open Images [16]. The use of these datasets enables the effective evaluation of the performance of object detection, classification, and segmentation algorithms. Although the kinds and quantities of these datasets are considerable, these datasets are mainly composed of natural scenes and network images and cannot be directly applied to precision agricultural visual tasks. In the study of the method of using computer vision technology to detect weeds, field weed image datasets are critical for the construction of an algorithm and the test of its effect. In fact, public plant image datasets that can be used for precision agriculture tasks should be based on plants or their leaves, but few public datasets meet this requirement [17]. Researchers face a series of problems, such as few databases and poor algorithm mobility. When researchers use different datasets for specific weed detection algorithms, evaluating different methods on the basis of the results of published literature is difficult or impossible. As computer vision and machine learning continue to impact agriculture, the number of public image datasets designated for specific agriculture tasks has gradually increased since 2015, effectively promoting the development of computer vision technology in precision agriculture. Table 1 lists several common datasets related to the field of weed detection and identification. Part of datasets contain leaf-level ground truth or pixel-level annotations, which can be widely used for weed detection, species identification, and leaf segmentation. The publication of increasing standard datasets will help further break the bottleneck of algorithm research on weed detection tasks.

**Figure 1:**
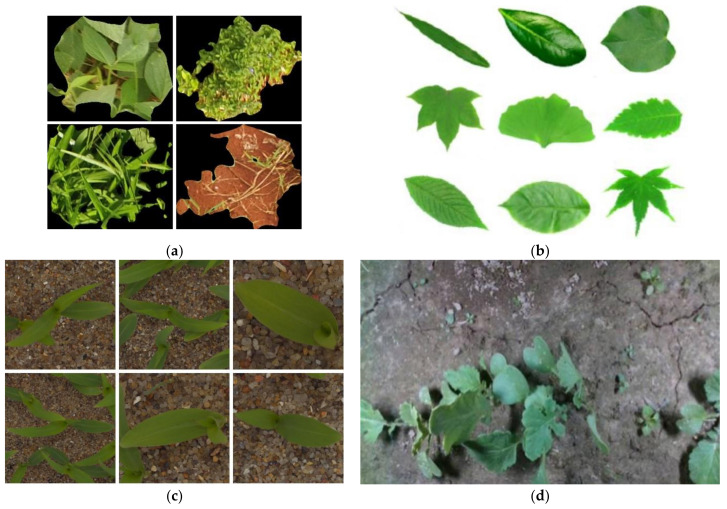
Four typical plant datasets: (**a**) Grass-Broadleaf database [19], with images of soybean, broadleaf weed, grass, and soil; (**b**) Flavia dataset [28]; (**c**) plant seedlings dataset [20]; (**d**) food crops and weeds dataset [26].

Figure 1 shows four typical plant dataset images, representing different situations: (a) demonstrates the images of a target plant segmented from a cluttered background, (b) presents plant leaves with a white background, (c) shows unsegmented maize, and (d) depicts crops and weeds on land.

Table 2 further compares the results of different methods under the same dataset. The comparison results of the three methods are listed under each typical dataset. It can be seen that with the continuous development of the algorithm, the accuracy is getting higher and higher.

## 3. Traditional Machine Learning Weed Detection Methods

In the early stage, many scholars used machine learning algorithms combined with image features to conduct weed recognition tasks, achieving the purpose of weed detection. These traditional ML methods require a small sample size and short training time; they also have a low requirement for graphics processing units. They can be used in agricultural machinery and equipment at a low cost, providing an effective method and approach for realizing plant identification and weed detection based on image-processing technology.

These intelligent technologies rely on the continuous development of machine vision technology. Machine vision technology uses a series of image-processing methods to extract the shallow features of weeds and then sends them to a classifier for detection. Initially, crops or weeds are identified by calculating the texture, shape, color, or spectral features of images. For example, Le et al. [38] realized the distinction between corn and single species of weeds on the basis of Local Binary Pattern (LBP) texture features and SVM. Chen et al. [39] proposed a multi-feature weed reverse location method in a soybean field on the basis of shape and color features. Zhu et al. [40] proposed a classification method for five kinds of weeds in farmland on the basis of shape and texture. Zhang et al. [41] conducted a comparative analysis of the gray distribution of each component in the color space of RGB, HSV, and HIS of common weeds in a field at the pea seedling stage. They proposed a method for weed segmentation and extraction in complex background based on R-B color difference features. Some scholars have used plant height [42] or location information [43,44,45] to improve the identification accuracy, but these methods are easily affected by vibration or other uncontrolled motion in practical application [46]. Moreover, some research has focused on using a single feature to identify plants, which has low accuracy and poor stability.

To deal with the problems of a complex field environment and the low accuracy and poor stability of a single feature, some scholars have also proposed to integrate multiple features to improve the accuracy. For instance, He et al. [47] integrated multisource recognition information of different features, such as plant leaf shape, fractal dimension, and texture. They combined the good classification and promotion capabilities of SVM in the case of small samples and the advantages of Dempster–Shafer evidence theory of incomplete and uncertain information. Compared with single-feature recognition, this multi-feature decision fusion recognition method has better stability and a higher recognition accuracy. Sabzi et al. [5] proposed a machine vision prototype based on video processing and meta-heuristic classifiers based on Gray-level Co-occurrence Matrix (GLCM), color feature, texture feature, invariant moment, and shape feature. They used them to identify and classify 4299 samples from potatoes and five weed species online, achieving high accuracy. Deng et al. [48] integrated the color, shape, and texture features of weed images with a total of 101-dimensional features to solve the problem of the low recognition accuracy of a single feature of weeds in a rice field. Tang et al. [44] used a combination of vertical projection and linear scanning in corn farms under different lighting conditions to identify the centerline of crop rows. This method only recognizes crop rows, all plants among rows are identified as weeds regardless of their type, and it is unsuitable for identifying different types of weeds. On the whole, these studies have provided effective methods and approaches for realizing plant recognition and weed detection based on image-processing technology in the early stage. However, most of the studies are only for the identification of different plant leaves rather than the precise detection of crops or weeds in a field. Few studies exist on the identification and location of plants and weeds in a complex practical background in a field, and the identification and detection of weeds in actual farmland require further research.

Table 3 lists some literature on the identification or classification of plant leaves by using traditional ML methods. These methods achieve their purpose in specific plant leaves and detection background, but they are unsuitable for large-scale rapid detection or classification of images in a natural environment.

Using drone images to classify vegetation and detect weeds on a large scale has become a hot spot. Object-Based Image Analysis (OBIA) classification has been replacing traditional classification methods like the pixel-based approach. The difficulty lies in setting the optimal combination of parameters. In order to solve this problem, Torres-Sánchez et al. (2015) [49] used unmanned aerial vehicle (UAV) images of different herbaceous row crops to develop an automatic thresholding algorithm under the OBIA framework, and research the influence of multiple parameters on vegetation classification, making the algorithm allow unsupervised classification. UAVs are less constrained by field conditions that may restrict the access and movement of operators or ground vehicle-based platforms, and can monitor weed areas on a large scale. Furthermore, UAV imagery offers high image resolution and high flexibility in terms of timing of image acquisition. The high image resolution allows detection of low weed densities. Therefore, such methods will have broad prospects in high-input agriculture.

### 3.1. Traditional Features and Their Advantages and Disadvantages for Common Weed Detection

Most of the traditional weed detection methods based on image processing utilize the feature differences between plant leaves and weeds to distinguish them. This article mainly discusses the traditional image features and their advantages and disadvantages for the detection and recognition of four features of weeds: texture, shape, spectrum, and color.

#### 3.1.1. Texture Features

Texture features are regional features that reflect the spatial distribution among pixels, which have been widely used in image classification [56,57,58]. Plant leaves are usually flat, and different leaves have diverse vein texture and leaf surface roughness information. The texture information can be used to distinguish crops and weeds effectively. Texture feature methods can mainly be divided into four categories: (1) statistical method, (2) structural method, (3) model-based method, and (4) transform-based method [59]. The most common texture feature descriptors used in weed detection include GLCM [60] and Gray-level Gradient Co-occurrence Matrix (GGCM) based on statistical texture analysis methods, LBP based on structural texture analysis methods, fractal dimension based on model methods, and Gabor based on transformation methods. The LBP feature can reflect the microstructure among pixels, and the improved LBP feature has the advantages of rotation and translation invariance. In essence, the Gabor feature has the effect of allowing the information of a certain frequency band to pass through it, and the remaining sub-information is filtered out. GLCM usually contains 10 statistics, which can reflect the spatial correlation of gray values of any two points in an image. GGCM considers the gradient information on the basis of GLCM, and it mainly has 15 statistics. The fractal dimension uses the self-similarity between local and whole research objects, and its methods include “blanket” algorithm, fractal Fourier, and box-counting dimension [61].

A large amount of texture information in crop and weed leaves plays an important role in recognition and classification tasks [62]. For example, Bakhshipour et al. [63] extracted 52 texture features (GLCM features in four directions) from wavelet multiresolution images for weed segmentation. Ishak et al. [52] used the combination of Gabor wavelet (GW) and gradient field distribution (GFD) to extract a new feature vector set based on directional texture features to classify weed species. Mustapha et al. [64] constructed a method based on texture feature extraction, which extracts texture features from field images composed of wide and narrow leaf weeds. However, these techniques cannot reliably and accurately perform classification tasks in complex natural scenarios, such as high weed density, overlapping, or obscured weeds and crops.

#### 3.1.2. Shape Features

Shape features play an important role in image analysis for weed detection. They mainly include shape parameters, region-based descriptors, and contour-based descriptors. Generally, shape parameters include 11 kinds: perimeter, area, diameter, minor axis length, major axis length, eccentricity, compactness, rectangularity, circularity, convexity, and solidity. These parameters are the most intuitive, easy to implement, and unaffected by lighting. Region-based descriptors include Hu moment invariants and two-dimensional Fourier descriptors (FDs). Hu moment invariants are a shape descriptor proposed by Hu (1962) [65]. They are a normalized function based on shape boundary and its internal region information and contain seven invariant moment parameters in total. They are independent of geometric translation, scaling, or rotation and are robust to noise. Two-dimensional FDs describe the shape region by establishing feature points in the region plane and carrying out Fourier transforms on rows and columns at the same time. Contexture-based descriptors mainly include spatial position descriptor, curvature scale descriptor, and one-dimensional FD.

These shape features have been successfully applied in the species recognition task of plant leaf images [66,67,68]. For example, Pereira et al. [69] used five shape descriptors, namely, beam angle statistics, FD, Hu moment invariants, multiscale fractal dimension, and Tensor Scale Descriptor (TSD), in shape analysis to describe the contour shape of aquatic weeds. Bakhshipour and Jafari [51] extracted four major shape factors, Hu moment invariants, and FDs to distinguish weeds and crops on different classifiers. Chen et al. [39] used eight shape features and Hu moment invariants combined with color features to detect weeds in a soybean field.

Different species of plants have distinct shape features, but the shape of the leaves can be distorted by disease, insects, and even human and mechanical damage. Most research is conducted under the ideal condition of specific leaves without background. In a field environment, problems of overlap or occlusion of plant leaves occur. Therefore, the task of weed identification is difficult to complete by only basing on shape features. They should be combined with other features to improve accuracy.

#### 3.1.3. Spectral Features

Spectral features are an effective method to distinguish plants with different leaf colors. When the spectral reflectance of weeds is remarkably different from that of crops [70], weeds and crops can be distinguished using spectral features. The spectral features are robust to partial occlusion and tend to decrease in calculation [71]. Some scholars have applied visible light and near-infrared spectra (Vis–NIR) [72,73], multispectral/hyperspectral imaging [74], and fluorescence [75] in the detection of different plants.

Pignatti et al. [76] distinguished corn crops and weeds by using the contents of chlorophyll and carotenoid retrieved using spectral indices or by inverting PROSAIL (coupled PROSPECT and SAIL radiative transfer models, [77]), as well as the species of weeds. Some scholars have also used Vis–NIR to classify weeds in crops, but studies are limited to laboratory feasibility studies and rely extensively on stoichiometry to select effective wavelengths and establish calibration models [78,79]. Elstone et al. [80] achieved good results in the identification of weeds and crops by using RGB and multispectral images in a lettuce field. However, weeds in plateau tropical conditions have different shapes and grow in large blocks, such that detecting them is difficult. Spectral sensors (spectrometers) can be used to measure the reflection intensity of multiple wavelengths and provide sufficient information to distinguish vegetation from soil. Nevertheless, they hardly distinguish species, especially in the early growth stages when crops and weeds have similar reflective characteristics [81,82].

During the growth and development stages of plants, the interaction between light and observed geometry and leaf angle distribution, as well as the variability of the spectral features of plant species, can affect hyperspectral detection. Capturing a multispectral image, hence, depends on the climatic conditions of the day, which changes the reflectivity of plants due to the amount of light absorbed. Although research on the identification of crop weeds by using sensitive spectral bands has achieved encouraging results, the accuracy is low under the condition that the spectral difference between crops and weeds is unobvious or the leaf reflection is affected by moisture, plant disease, growth period, and other factors [83]. Therefore, a combination of multiple features, such as shape and texture features, should be considered [84].

#### 3.1.4. Color Features

The accuracy of color-based detection highly depends on the plant being studied and its color differences. Color is insensitive to the adjustment of scale, size, and position. In addition, it can provide information about unusable objects. It is a common method used to segment plants from the background by using the difference in color features. Hamuda et al. [85] summarized the advantages and disadvantages of plant segmentation for color index-based methods. Tang et al. [86] proposed to modify the color component (2G−R−B) and use the excessive green component ExG=2G−R−B of the RGB color space to segment. Ghasab et al. [87] and Zhao et al. [88] used the color moments of the RGB color space (including mean, standard deviation, and skewness) to represent the color features of plant leaves. Rasmussen et al. [89] used the color difference between green weeds and senescent cereals to propose a simple, semi-automatic, and robust procedure for weed detection in pre-harvest cereals, which has strong practical significance.

In addition, R, G, and B components have a high degree of correlation, which is suitable for color display but not for segmentation and analysis [90]. Therefore, many methods transform images from the RGB color space to other color spaces, such as HIS, HSV, Lab, and YCrCb. Tang et al. [44] used the YCrCb color space $Cg\;\left(Cg=G-y\right)$ to describe the green features of green crops under different illumination conditions. Hamuda et al. [91] believe that the HSV color space is more in line with human color perception than other color spaces and has strong robustness to illumination changes. The HSV color space was used to distinguish weeds, soil, and other residues in cauliflower fields under actual field conditions. Guo et al. [92] utilized 18 color features (r, g, b; Y, Cb, Cr; H, S, L; H, S, V; L*, a*, b*; L*, u*, v*), which were defined in 6 color spaces (RGB, YCbCr, HSL, HSV, CIEL*a*b*, and CIEL*u*v*). Knoll et al. [93] and Jin [94] also utilized different color spaces.

Color is the most unstable feature used for plant identification. When the color difference is unobvious, color-based methods may not be able to distinguish weeds from crops accurately. These methods can be affected by leaf disease, plant seasonal changes in color, or different lighting conditions. Table 4 compares the advantages and disadvantages of four common image features for weed detection.

### 3.2. Multi-Feature Fusion

The similarity between weeds and crops makes using a single image feature to detect weeds and crops almost impossible. The commonly used image features can achieve the purpose of weed detection, but the experimental accuracy is low and the stability is poor in a nonideal environment due to the complex interference factors in the actual field. Table 4 indicates that the four features are from different perspectives and complement one another in function. To improve the experimental accuracy, researchers have successively used the method of multi-feature fusion to solve the problem of weed detection.

Ghazali et al. [95] combined statistical GLCM, structural method fast Fourier transform, and scale-invariant feature transform and achieved more than 80% accuracy in the real-time weed control system of an oil palm plantation. Li et al. [96] used a method based on the combination of shape analysis and spectral angle matching to identify weeds in watermelon fields. Shape and spectral features were used separately, excluding texture features. Chowdhury et al. [97] focused on vegetation classification on the basis of features extracted from a local binary model and GLCM and classified images in accordance with the density of grass to highlight the images with potential fire risks on both sides of the road. Tang [98] constructed a leaf texture feature extraction algorithm based on GGCM and an improved leaf color feature extraction algorithm combining K-means and SVM for plant leaf recognition. However, the problems of extracting leaf images and performing threshold segmentation under a complex background remain. He et al. [47] extracted three types of features of plant leaf shape, texture, and fractal dimension on the basis of field plant image processing. Compared with single-feature recognition, the multi-feature decision making fusion recognition method has better stability and higher accuracy, but it does not analyze the problem of feature selection. Chen et al. [99] studied the method of multi-feature fusion based on field weed detection at the corn seedling stage to analyze the selection of common feature descriptor combinations. On the basis of 6 feature descriptors commonly used in recent years (rotation-invariant LBP, HOG, GLCM, GGCM, Hu moment invariant, and Gabor), 18 multi-feature groups were formed. The combination of rotation-invariant LBP feature and GGCM showed the highest accuracy. Experiments have also proven that the average accuracy of multi-feature fusion is not necessarily higher than that of single-feature fusion. Nursuriati et al. [100] used three single features, namely, shape, color, and texture, or fusion features of Malaysian herbal plant leaves for identification experiments. The experimental results showed that when the three features were fused, the average accuracy was highest, followed by the average accuracy when using only the texture features. When shape features are combined with texture features, the average accuracy decreased. Lin et al. [101] studied the feasibility of integrating spectral, shape, and texture features to identify corn and seven kinds of weeds. They found that from the perspective of accessibility of crop/weed discriminant features, spectral and shape features can be used as the optimal features to develop weed identification. Nonetheless, such a method has not been applied in a complex natural environment, and the method needs further research. Yang et al. [37] proposed a new shape feature, MTD, which was combined with the LBP–HF texture feature for leaf classification and retrieval tasks. This method is efficient and suitable for large-scale plant species identification. However, its features should be designed manually and cannot be learned automatically, and other important features of leaves are not utilized.

In conclusion, these multi-feature fusion methods can solve the problem of weed detection and improve the accuracy of experiments, but some problems have not been completely solved. For example, for many interference factors under nonideal conditions, the accuracy and stability of experiments should be further improved.

### 3.3. Classifier

SVMs and Artificial Neural Networks (ANNs) have been widely used in crop and weed classification [102,103]. SVMs can solve the problems of nonlinear and high-dimensional pattern recognition and have good performance in dealing with small-sample problems and nonlocal minimum problems. ANNs have a strong learning capability and can classify untrained data [63]. Other algorithms often involved in the literature include K-nearest neighbor (KNN) [104] and random forest [105,106], naive Bayesian algorithm [107,108], Bayesian classifier [109], and AdaBoost [110,111].

In recent years, relevant scholars have continued to study the use of various classifiers to identify and classify weeds. For instance, Jeon et al. [112] used a weed detection and image-processing algorithm based on ANN to distinguish weeds and crops in the soil background under uncontrolled outdoor light. Chen et al. [113] used an improved KNN weed image classification method combined with GW and regional covariance Lie group structure to classify four kinds of broad-leaved weed images. The overall recognition accuracy was 93.13%. Ahmed et al. [84] used SVM to identify 6 weeds in a dataset of 224 images, and the optimal combination of its extractor could achieve 97.3% accuracy. Rumpf et al. [114] proposed a sequential classification method and used three different SVM models to distinguish not only weeds and barleys but also weeds of monocotyledon and dicotyledon plants.

Some literature has utilized multiple classifiers. For example, Bakhshipour and Jafari [51] evaluated the performance of using SVM and ANN based on shape features in accordance with the detection problem of four common weeds in sugar beet fields. The results showed that the overall accuracy of SVM was 95.00%, higher than that of ANN (i.e., 92.92%). Miao et al. [115] proposed a method based on image segmentation and reconstruction to solve the problems of low recognition accuracy and invalid shape feature in the recognition process of overlapping leaves. The recognition results in different classifiers, such as SVM, KNN, DT, and naive Bayes, were compared using 78-dimensional features, such as color features, LBP texture features, and fractal box dimensions. The best was SVM. Ashraf et al. [116] developed two kinds of rice field image classification technologies based on the density of weeds. The first method was to use GLCM combined with SVM to achieve a precision of 73%, and the second method was to use invariant scale and rotation moment based on a random forest classifier to achieve a precision of 86%. The limitation of the two methods is that they do not target other types of weeds, such as broadleaf weeds and sedges. Pantazi et al. [117] implemented a machine vision-based method that can identify 10 types of weeds, including corn plants and specific species. This method uses a Gaussian classifier, a self-organizing feature map (SOFM), an SVM, and an autoencoder as the four hybrid classifiers. However, this method can only recognize four weeds with a maximum accuracy of over 90%. When applied in the field, the system error is relatively large.

In summary, scholars have focused on improving classifiers based on machine vision or the corresponding image features of plants, which is of great significance to improve the accuracy. They can utilize the sample features in the case of small samples and do not require high hardware. They are conducive to practical deployment and play an important role in weed identification or classification in common scenes.

## 4. Weed Detection and Identification Methods Based on Deep Learning

The great progress and popularization of image-capturing devices have made capturing images easy. Meanwhile, the cost of computer hardware has been greatly reduced, and the computing power of GPU has been remarkably improved. Deep learning has been extended to the agricultural field [118,119,120]. Methods based on deep learning have achieved good results in weed detection and classification [121]. Although traditional ML methods are easy to understand and many improvements have been made, most of them are verified in low-density images. Occlusion, clustering, and changing lighting conditions in a natural environment remain major challenges in detection and localization [122].

Deep learning has a unique network feature structure, and features extracted using various deep learning methods are more effective than manually extracted features. Higher-level features can be obtained by learning local features from the bottom and then synthesizing those features from the top. Diverse features at different levels can correspond to various tasks. In the field of weed detection, deep learning methods use spatial and semantic feature differences to realize the identification and detection of crops and weeds and effectively improve the accuracy of weed identification and detection. In recent years, commonly used deep learning networks to solve the problem of weed detection include CNNs and fully convolutional networks (FCNs). Various methods in semi- and unsupervised fields have also emerged to reduce the labeling cost. In many cases, classification results obtained using these deep learning algorithms are better than those generated using traditional algorithms [123]. The use of traditional algorithms to classify different types of crops with high accuracy is still difficult. Deep learning methods need to rely on a large number of datasets for training, and the difficulty of collecting crop and weed images also demonstrates the disadvantages of deep learning methods for weed identification.

### 4.1. Weed Detection and Identification Methods Based on CNNs

CNNs are increasingly used in weed detection, and methods based on deep CNNs have achieved good results in weed detection and classification. For instance, Dyrmann et al. [124], Yu et al. [125], and Olsen et al. [21] used such methods. Potena et al. [126] adopted two different CNNs to process RGB and NIR images to identify crops and weeds rapidly and accurately. A lightweight CNN was used for fast and robust vegetation segmentation, then a deeper CNN was used to classify the extracted pixels between crops and weeds. Beeharry and Bassoo [127] evaluated the performance of two weed detection algorithms based on UAV images, ANN and AlexNet. The experimental results showed that the accuracy of AlexNet in weed detection was more than 99%, whereas the accuracy of ANN on the same dataset was 48%. Ramirez et al. [128] established an aerial image weed segmentation model and compared it with SegNet and U-Net. The research results showed that the data balance and better spatial semantic information made the experimental results more accurate. Patidar et al. [129] proposed an improved Mask RCNN model to extract early cranesbill seedlings. These weeds can be used as herbal medicines for rheumatic disease. The proposed method enabled the weeds to be completely separated from the original image to obtain complete nutrients and increase yield. You et al. [130] proposed a semantic segmentation method for weed crop detection based on deep neural networks (DNNs). Four additional components were integrated to improve the segmentation accuracy, which provided enhanced performance for weeds of arbitrary shape in a complex environment. These methods do not rely on image preprocessing and data conversion and can independently obtain useful feature information in images. The recognition accuracy is better than that of manually designed features under traditional ML methods.

CNN frameworks, such as AlexNet [19], ResNet [131,132], VGG [133], Google [134], U-Net, MobileNets, and DenseNet [135], are also widely used in weed detection. These methods stand out from other conventional index-based methods. For example, Chechliński et al. [135] measured four different plants in diverse growing places and light conditions, and their custom framework combined U-Net, MobileNets, DenseNet, and ResNet.

### 4.2. Weed Detection and Identification Methods Based on FCNs

FCNs are algorithms that automatically learn features and implement forward and reverse processes in an end-to-end manner. In recent years, FCNs have made great achievements in computer vision [136] and remote sensing applications [137,138]. Dyrmann et al. [139] proposed a method to detect weeds in color images automatically by using an FCN under severe occlusion. Huang et al. [140] captured a high-resolution UAV image over a rice field and adopted an FCN for pixel-level classification. Ma et al. [25] proposed a SegNet semantic segmentation method based on FCNs for the problem of weed detection in rice fields. Compared with the classic FCN model and U-Net model, the proposed method exhibited significantly higher accuracy and could effectively classify the pixels of rice seedlings, background, and weeds in rice field images. To control weeds in the early stages of growth, Fu et al. [141] proposed a segmentation method based on FCNs for high-resolution remote sensing images. On the basis of the VGG16 CNN model, a pretrained FCN was used to fine-tune the object data. This method could effectively improve the segmentation effect. FCNs were used to solve semantic-level image segmentation and pixel-level classification of images, which further developed the problem of weed segmentation. However, this method only classified each pixel without considering the relationship among pixels.

### 4.3. Weed Detection and Identification Methods Based on Semi- and Unsupervised Feature Learning

Supervised deep neural networks rely on artificially annotated data; even with the use of rotation and cropping data enhancement techniques, at least hundreds of annotated images are still required for supervised training. Relevant scholars began to study semi-supervised learning with only a small amount of labeled data and unsupervised feature learning without data labeling [142,143]. Hu et al. [34] proposed a new image-based deep learning architecture called Graph Weed Network (GWN). The purpose is to identify multiple types of weeds from RGB images collected from complex pastures. GWN can be regarded as a semi-supervised learning method, which alleviates the complex annotation task. The evaluation on the DeepWeeds dataset reached the highest accuracy of 98.1% at the time. Jiang et al. [144] proposed semi-supervised GCN–ResNet101 to improve the recognition accuracy of crops and weeds in a limited labeled dataset, combining the advantages of CNN features and the semi-supervised learning capability of the graph. Tang et al. [145] combined k-means unsupervised feature learning with the advantages of multilayered and refined CNN parameters as a pretraining process for the identification of weeds in soybean seedlings. This method replaces the random initialization weights of traditional CNN parameters, which effectively proves that this method is more accurate than randomly initialized convolutional networks. Bah et al. [146] proposed an automatic learning method for weed detection in the UAV images of bean and spinach fields, which was based on CNN and an unsupervised training dataset. Experimental results proved that the performance of this method was close to that of supervised data labeling. Ferreira et al. [33] tested two latest unsupervised deep clustering algorithms by using two public weed datasets. They proposed to use semiautomatic data labeling for weed identification. Compared with manually marking each image, semiautomatic data labeling could reduce the marking cost by hundreds of times. Then, NMI and unsupervised clustering accuracy indexes were used to evaluate the performance of pure unsupervised clustering. The use of unsupervised learning and clustering on agricultural issues will continue to be the direction of continuous development.

### 4.4. Other Deep Learning Methods

Researchers have proposed various other deep learning methods to solve the problem of weed detection and achieved good results. For example, Sadgrove et al. [147] proposed the Color Feature Extreme Learning Machine (CF-ELM). It is an implementation of the Extreme Learning Machine (ELM, which is a single-layer feed-forward neural network. It has a partially connected hidden layer and a fully connected output layer and uses three color inputs instead of the standard grayscale input. The authors used the inputs in three different color systems of HSV, RGB, and Y’UV to test and compare the accuracy and time consumption with those of the standard grayscale ELM. The proposed method performed well on three datasets: weed detection, vehicle detection, and population detection. It is highly suitable for use in agriculture or pastoral landscape. Abdalla et al. [148] compared three transfer learning methods based on VGG16 for semantic segmentation of high-density weed and oilseed rape images. Annotated images were trained end to end through the extensive use of data enhancement and transfer learning. The fine-tuning was based on the VGG16 encoder for feature extraction, and shallow machine learning classifiers were used for segmentation. Raja et al. [149] proposed a real-time online weed detection and classification algorithm based on crop signal for lettuce. The spraying mechanism was combined with a machine vision system to realize the classification task in the case of high weed density and achieve the purpose of precise spraying of weeds with herbicides. Khan et al. [150] proposed a small-cascaded encoder-decoder (CED-NET) architecture to distinguish crops from weeds, in which each level of the encoder and decoder network was independently trained for crop or weed segmentation. This network was compared with other state-of-the-art networks in four public datasets. The experiment proved that it was superior to U-Net, SegNet, FCN-8s, and DeepLabv3.

All in all, in order to further compare deep learning methods. Table 5 summarizes the five architectures and comparison experiment group. The five frameworks are Convolutional Neural Networks, Regional Proposal Networks, Fully Convolutional Networks, Graph Convolutional Networks, and Hybrid Networks. The order of comparison experiment accuracy is the order in “Comparison group”. Among them, Osorio et al. only gave “Precision” but not “Accuracy”. This is different from the calculation formula of “Accuracy”. The specific calculation formula needs to check the current work is classification recognition or semantic segmentation. Researchers could refer to the review written by Hasan et al. [12], which described 23 evaluation metrics by different researchers of the related works.

## 5. Weeding Machinery

In addition to the intelligent detection of weeds based on computer vision technology and to achieve spraying the target variable, autonomous agricultural robots that continuously improve the accuracy and efficiency have also been widely used in weeding fields. Researchers have relied on powerful computer vision and mechanical techniques to design various fully automated weed control robots. Robotic weeding uses computer vision to detect crops and weeds and selectively applies herbicides to the detected weeds [133] or eliminates weeds among rows [153,154] to achieve the purpose of precision agriculture. Raja et al. [155] proposed a weed knife control system based on a robot vision-based 3D geometric detection algorithm. Corresponding mechanical knife devices were also designed for automatic control of weeds in tomato and lettuce fields, which could work efficiently in a high-weed density environment. The system proposed by Kounalakis et al. [123] was mainly used to detect a specific plant on grassland, which would cause health, yield, and quality problems if eaten by animals. The implementation of this method relied on the design of a robot platform that could accurately detect the plant. The research of Chechliński et al. [135] mapped a weeding device, which would be installed behind a tractor, and the weeding tool would be installed behind a camera. The weeding tool could be replaced with insecticide or steam nozzles. Compared with traditional methods, intelligent weeding machines and equipment save manpower, are efficient, and can increase productivity. The future development direction of agricultural machinery will be to develop more efficient and multitask automatic machinery and equipment.

## 6. Discussion

### 6.1. Various Weed Detection Tasks

**Figure 2:**
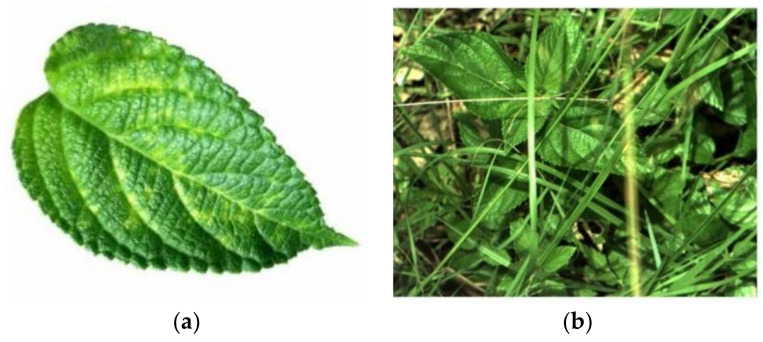
Plant leaves in different backgrounds: (**a**) is the plant leaf image taken in a controlled laboratory environment; (**b**) is the plant leaf image obtained from the Deepweeds dataset [21], which is shot on-site to capture the true view of the whole plant).

The tasks of weed detection are diverse. Through literature analysis, they are mainly reflected in the following aspects:

(1) Different crops and weed species and diverse problems for the detection of various species. When a crop is similar to associated weeds, the detection is difficult. Relevant research has only classified and identified the leaves of specific plants rather than actual field images in a complex background, as shown in Figure 2. When applied to weed detection in the field, the accuracy is low and the stability is poor.
(2) Different datasets and evaluation indicators. At present, few public datasets are available. Consequently, many studies have been conducted on the basis of self-built datasets. Even if the main body of some datasets is the same crop, the portability of the algorithm is poor under different growth periods, illumination, and actual field backgrounds. Relevant evaluation indicators are not comparable due to the different basis of the dataset developed by the algorithm. The actual performance is difficult to determine.

### 6.2. Multiple Complex Factors Affect Weed Detection

The natural properties of weeds are complex, with a wide variety of species, wide distribution, numerous leaf shapes and sizes, and random growth, forming various texture features. In the bud stage of weeds, most plants are small in size, vary in appearance, and have high germination density. As a result, accurate statistics is difficult to perform. The main factors affecting the performance of weed detection are as follows:

(1) The influence of different growth stages. Most plants change their leaf morphology, texture, and spectral characteristics in different seasons or growth and development stages.
(2) The influence of changing light conditions. When light conditions are different, the shade of the plant canopy and the angle of the sun will affect the color of the vegetation. Some scholars have used the ultra-green index and the Otsu algorithm to solve the problems caused by ambient light. In particular, Atrand et al. [156] solved the problems by using camera filters and different types of cameras. HIS color model was also applied, and grayscale images with H component were generated to reduce the impact of uneven lighting on color images [157].
(3) Influence of overlapping leaves and occlusion. The accurate segmentation of plants is a challenging task. In complex actual field images, overlapping leaves, occlusions, leaf shadows, dead leaves, and damaged leaves will make it impossible to segment the leaves effectively when processing the images.
(4) Bottleneck of weed detection. Factors, such as hardware, algorithm complexity, and plant density, limit the actual detection speed or accuracy. Hence, fast image processing and accurate weed identification remains extremely important challenges.

## 7. Summary and Outlook

This article reviews the work of researchers using traditional machine learning and deep learning methods in computer vision technology in recent years. Four traditional characteristics and their advantages and disadvantages in traditional ML methods are analyzed. The respective characteristics of related work based on deep learning algorithms are introduced. Related public datasets and weeding machinery are also presented. Lastly, the future work of weed detection is prospected. In the past two decades, weed detection has made great progress. On the basis of traditional machine learning methods and deep learning-based weed detection methods, high levels of automatic weed detection and weeding have been achieved using various platforms and mechanical equipment. These methods have laid a good foundation for achieving high efficiency and precise weeding in the future. In the future, weed detection and related fields will have the following development trends:

(1) Further research on semi- or unsupervised feature learning will be a hotspot of weed detection in the future. Researchers have obtained good results in diverse specific background, but they still lack generality and robustness. Deep learning-based methods show an encouraging promise, but the large number of labeled samples increases the manual requirements. The verification and comparison of new development algorithms also require sufficient sample size and corresponding ground truth datasets. Compared with various weeds, field crop images are relatively easy to obtain. In view of the above reasons, weed detection methods based on semi- or unsupervised feature learning will continue to be a popular research topic in the future.
(2) With the use of the technology of weed detection and accumulation to develop an automatic crop guidance system, agricultural operations can be carried out in various aspects, such as harvest, weeding, spraying, and transportation. Automatically guided agricultural vehicles do not fatigue and reduce the labor intensity of the operator, thus improving efficiency and safety. However, at present, few methods and devices meet the high requirements of practical applications. Considerable work should be done to develop equipment with high performance and cost efficiency.
(3) Traditional and deep learning methods have their own advantages. In the future, the advantages of the two methods should be fully utilized for further research. To improve the level of weed detection and weeding, solutions have been proposed to solve the difficult practical problems, such as plant illumination, overlapping leaves, occlusion, and classifier or network structure optimization.

## Figures and Tables

**Table 1:** Public weed image datasets and their features.

Reference	Datasets	Purpose	Plant	Image Size	Features
[18]	Perennial ryegrass and weed	Weed detection and control	Dandelion, ground ivy, spotted spurge, and ryegrass	1920 × 1080 33,086	It includes 17,600 positive images (contain target weeds) and 15,486 negative images (contain perennial ryegrass with no target weeds).
[19]	Grass-Broadleaf	Weed detection by using ConvNets	Soil, soybean, broadleaf, and grass weeds	4000 × 3000 15,336	Data are from a set of images captured using a UAV and the SLIC algorithm. These images are segmented, and the segments are annotated manually. The ratio of soil: soybeans: grass: broadleaf weeds is roughly 3:7:3:1 (Figure 1a).
[20]	Plant seedlings dataset	Identifying plant species and weeding in the early growth stage	12 weed and crop species of Danish arable land	5184 × 3456 407	Each image is provided with an ID and associated with a single species. The dataset contains a full image, automatically segmented plants, and single plants that are not segmented.
[21]	DeepWeeds	Classification of multiple weed species based on deep learning	8 nationally significant weed species native to 8 locations across northern Australia	256 × 256 17,509	Each class contains between 1009 and 1125 images of the corresponding species, with a total of over 8000 images of positive species classes.
[22]	Open Plant Phenotype Database	Plant detection and classification algorithms	47 species of common weeds in Denmark	1000 × 1000 7590	It includes 47 different species of monocotyledonous and dicotyledonous weeds in arable crops in Denmark. Several plant species were cultivated in a semifield setting to mimic natural growth conditions.
[23]	WeedNet	Dense semantic classification, vegetation detection	Crops and weeds	/ 465	Three kinds of multispectral image datasets are included: one contains only 132 images of crops, the other has 243 images of weeds, and the third one contains 90 images of crop–weed.
[24]	Sugar beet	Plant classification, localization, and mapping	Sugar beets and 9 different types of weed	1296 × 966 >10,000	Data were recorded 3 times per week until the field was no longer accessible to the machinery without damaging the crops. The robot carried a four-channel multispectral camera and an RGB-D sensor.
[25]	Rice seedlings and weeds	Image segmentation of rice seedling and weeds	Rice seedlings and weed background	912 × 1024 224	The images were selected in the paddy fields, and all weeds were in early growth stages. The data sample included GT and RGB images (Figure 1c).
[26]	Food crops and weed	Crop and weed identification	6 food crops and 8 weed species	720 × 1280 1118	Datasets of 14 basic food crops and weeds in controlled environment and field conditions at different growth stages and manually annotated images are included (Figure 1d).
[27]	Crop and weed	Instance segmentation for fine detection	Maize, the common bean, and a variety of weeds	1200 × 2048 2489	The crops include maize and the common bean. Weeds include cultivated and natural weeds. Each mask is annotated with the species name of the plant.
[28]	Flavia	Plant leaf classification	Leaves of 32 plants	1600 × 1200 1907	Each plant has a minimum of 50 leaves and a maximum of 77. The background of the leaf image is white (Figure 1b).
[29]	CropDeep	Crop classification and testing	30 common vegetables and fruits	1000 × 1000 31,147	At least 1100 annotated samples per category and vegetables or fruits with different parts and periods of growth are included. A high degree of similarity exists among certain categories in the dataset.

**Table 2:** Comparison of different methods under the same typical dataset.

Reference	Dataset	Method	Evaluation Metrics
Chavan et al. (2018) [30]	Plant seedlings dataset [20]	AgroAVNET (A hybrid model of AlexNet and VGGNET)	Accuracy: 98.23%
Trong et al. (2021) [31]		Yielding multi-fold training (YMufT) strategy and DNN; Min-class-max-bound procedure (MCMB); Resnet	Accuracy: 97.18%
Xu et al. (2021) [32]		Depthwise separable convolutional neural network, Xception	Accuracy: 99.63%
Olsen et al. (2019) [21]	Deepweeds [21]	Dataset was classified with the ResNet-50 and Inception-v3 CNN models to establish a baseline level of performance for comparison.	Accuracy: 95.1% (Inception-v3) Accuracy: 95.7% (ResNet-50)
Ferreira et al. (2019) [33]		Joint Unsupervised Learning of Deep Representations and Image Clusters (JULE) and Deep Clustering for Unsupervised Learning of Visual Features (DeepCluster)	Precision: 95%
Hu et al. (2020) [34]		GWN (Graph Weeds Net)	Accuracy: 98.1%
Naresh et al. (2016) [35]	Flavia [28]	MLBP (Modified Local binary patterns)	Accuracy: 97.55%
Mahajan et al. (2021) [36]		Support vector machine with adaptive boosting	Precision:95.85%
Yang C. Z. (2021) [37]		MTD (multiscale triangle descriptor) and LBP-HF (local binary pattern histogram Fourier)	Accuracy: 99.1%

**Table 3:** Research status and problems of traditional machine learning methods.

Reference	Year	Purpose	Accuracy	Problems
[50]	2016	Combining HOG feature with Support Vector Machine (SVM) to identify grape leaves	83.50%	Single-feature detection has poor stability and low accuracy.
[35]	2016	Identifying different plant leaves on the basis of improved LBP	79.35%	
[51]	2018	Using three shape features to compare the effect of SVM or Artificial Neural Network (ANN) on detecting sugar beets and weeds	93.33%	Analysis on the selection of features is lacking.
[52]	2009	Combining GW (Gabor wavelet) and GFD (gradient field distribution) to classify different weeds	93.75%	
[53]	2015	Combining Gabor and Grey-level Co-occurrence Matrix (GLCM) to classify 31 plant leaves	91.60%	No actual field images are included, and the dataset is only composed of different plant leaves, without complex background, such as soil.
[54]	2017	Extracting the shape and texture features of an image to classify and recognize plant leaves	92.51%	
[55]	2015	Using improved LBP and GLCM to categorize fresh tea in the production line	94.80%	Nonwhole plants are detected and recognized, and only the same kind of leaves is classified.

**Table 4:** Comparison of the advantages and disadvantages of four common features.

Features	Advantages	Disadvantages
Texture	Has high accuracy, strong adaptability, and robustness	Grey-level co-occurrence matrix (GLCM takes a long time and does not meet the real-time processing requirements.
Shape	Independent of geometric translation, scaling, or rotation; robust to noise	Shapes are deformed by disease, insect eating, and man-made or mechanical damage and incomplete under overlap and occlusion.
Color	Insensitive to the adjustment of proportion, size, and position	Crops and weeds with similar color will fail; leaf lesions and plant seasonality will change color.
Spectral	Robust to partial occlusion	Spectral features vary in different growth stages of plants, are easily affected by the collection environment, and are unstable.

**Table 5:** Comparison of the typical deep learning methods.

Ref.	Crop	Types	Architecture	Strengths	Comparison Group	Highest Accuracy
[151] (2019)	Not specified	RGB	Convolutional neural network	Propose a low-cost Weed Identification System (WIS) using RGB images taken by drones as training data and applying CNN to build the identification model.	1.CNN-WIS 2.LBP 3.HOG	98.8% (CNN-WIS)
[152] (2020)	Lettuce	Multis-pectral	Region proposal network	A false green image was generated, which is the union of the red, green, and near infrared bands, in order to highlight the vegetation.	1.Mask R-CNN 2.HOG-SVM 3.YOLOv3	(Precision) 98% (Mask R-CNN)
[25] (2019)	Rice	RGB	Fully convolutional network	Proposed a SegNet semantic segmentation method based on FCN. Could effectively classify the pixels of rice seedlings, background, and weeds in rice field images.	1.SegNet 2.FCN 3.U-Net	92.7% (SegNet)
[144] (2020)	Corn, lettuce, radish	RGB	Graph convolutional network	Used GCN combined with state-of-the-art pre-training network (AlexNet, VGG16 and ResNet-101) to conduct comparative analysis on four datasets.	1.GCN-ResNet101 2.GCN-VGG16 3.GCN-AlexNet	97.8% (GCN-ResNet101)
[30] (2018)	Maise, common wheat, sugar beet	RGB	Hybrid Network	AgroAVNET is a hybrid model of AlexNet and VGGNET. The performance is compared with AlexNet, VGGNET and their variants and existing methods.	1.Hybrid Network (AgroAVNET) 2.VGGNet 3.AlexNet	98.23% (Hybrid Network)

## Data Availability

Not applicable.
